# Mind the tributary of the canal: Are stents necessary for insulinoma enucleation in proximity to a prominent Duct of Santorini: A case report and literature review

**DOI:** 10.1097/MD.0000000000031211

**Published:** 2022-10-28

**Authors:** Tiantong Liu, Qiang Xu, Xi Zou, Liang Zhu, Yupei Zhao

**Affiliations:** a Department of General Surgery, Peking Union Medical College Hospital, School of Medicine, Tsinghua University, Beijing, China; b Department of General Surgery, Peking Union Medical College Hospital, Beijing, China; c Chinese Academy of Medical Sciences and Peking Union Medical College, Beijing, China; d Department of Radiology, Peking Union Medical College Hospital, Beijing, China.

**Keywords:** enucleation, insulinoma, POPF, the Duct of Santorini

## Abstract

We describe a case of insulinoma located extremely close to the accessory pancreatic duct (APD), but away from the main pancreatic duct (MPD). Previous studies showed insulinoma enucleation is a safe procedure for small benign tumors >3 mm distant from the MPD. However, in this case enucleation of the tumor led to unanticipated APD injury and grade B post-operative pancreatic fistula (POPF). We provide detailed records of clinical management and argue that enucleation of tumors near APD needs to be carefully weighed.

**Patient concerns:**

The patient experienced a sudden increase of abdominal drain fluid and prolonged drainage time after a regular insulinoma enucleation surgery.

**Diagnosis:**

APD damage during the enucleation.

**Interventions:**

Drain fluid amylase concentration were regularly recorded and prolonged somatostatin analogs were administered.

**Outcomes:**

Amount of abdominal drain gradually decreased and the drain tube was removed on postoperative 37.

**Lessons:**

Benign pancreatic tumor close to the APD need to be evaluated carefully and clinical evidence is warranted to affirm the necessity of placing a pancreatic duct stent before the surgery.

## 1. Introduction

Insulinoma is the most commonly diagnosed functional pancreatic neuroendocrine tumor, and surgical removal is recommended in almost all guidelines.^[1–3]^ Small, benign insulinomas are readily curable by parenchymal-sparing pancreatic resection or enucleation, preserving the pancreatic parenchyma to the greatest possible extent and avoiding long-term pancreatic insufficiency.^[4–7]^ Patients typically have satisfactory clinical outcomes.^[4,8,9]^ However, occasions exist where a more extensive procedure is required when enucleation is difficult to perform.^[4,9]^ In particular, when the tumor is located only a few millimeters away from the main pancreatic duct (MPD), enucleation can sometimes lead to injury, causing persistent postoperative pancreatic fistula (POPF), one of the most common complications of pancreatic surgeries.^[10,11]^ Our group previously reported that a distance from the lesion to the MPD ≤ 2 mm was an independent risk factor for clinically-relevant POPF in patients with insulinoma; therefore, preoperative pancreatic duct stenting may be beneficial to these patients.^[12]^

Nevertheless, precautions, such as pancreatic stenting, are considered to lower the risk of POPF.^[13]^ Pancreatic duct stenting has been in use for decades. Some groups have reported that prophylactic pre-operative pancreatic stenting by endoscopic retrograde cholangio-pancreatography (ERCP) can facilitate the identification of MPD intraoperatively, simplify the resection of the target lesion, and protect the ductal system against iatrogenic injury.^[14]^ At the same time, stenting guarantees postoperative drainage of pancreatic fluid into the gastrointestinal tract, avoiding leakage from the pancreatic wound in cases of enucleation and partial resection of the pancreas.^[14]^

In fact, MPD is not the only “guest” and we need to treat it gently. In approximately 60% of patients, the structure of the pancreatic duct system remains similar, with the duct of the Wirsung fusing with the dorsal duct to become the primary continuation of the MPD, while the duct of Santorini remains functional but has a small diameter, draining into the minor duodenal papilla. Therefore, although the duct of Wirsung is clearly visualized on magnetic resonance cholangiopancreatography (MRCP) and 3-dimensional computed tomography (CT) image reconstruction, the duct of Santorini, like most side branches of the MPD, is barely visible or not seen at all for most patients.^[15]^ To make things worse, stenting of the duct of Santorini is usually impossible because it is too thin to reach by ERCP. Here, we present a case of successful enucleation of an insulinoma with the expected occurrence of POPF due to its proximity to the duct of Santorini and possible injury of the duct. We did not place a pancreatic stent preoperatively because of the limited expected benefit and iatrogenic injuries. Although we did not observe evident pancreatic duct leakage immediately upon removal of the tumor during the surgery, grade B POPF was recorded. Although the POPF lasted over a month, we observed a decline in the abdominal drainage fluid around POD (Postoperative day) 10, and the patient did not show severe symptoms that required extra intervention. Combined with our previous study, we further discuss the safety of enucleation of tumors with similar locations and the need for pre-operative pancreatic stenting when the expected risk of POPF is high.

## 2. Case report

**Figure 1.**
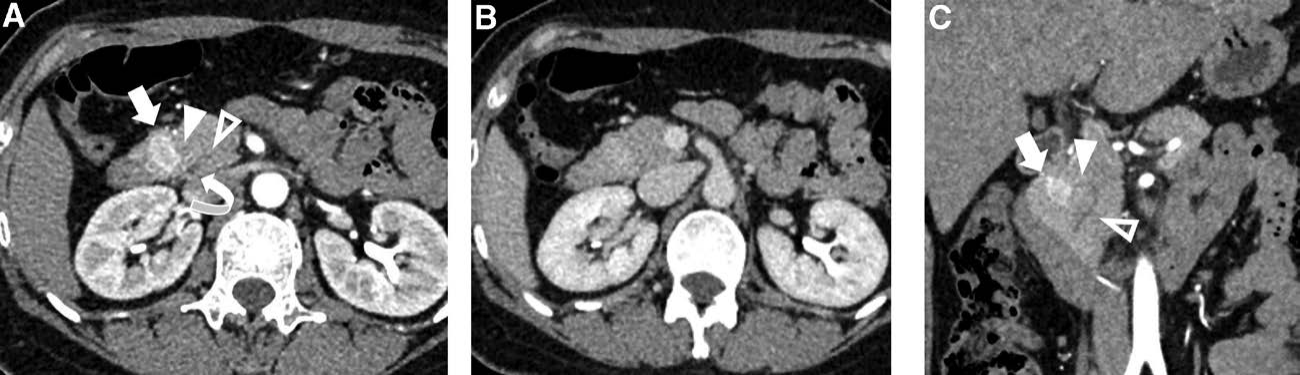
Contrast-enhanced CT “pNETs protocol.” (a) Axial early arterial phase image of 1-mm thickness revealed a hyper-enhancing nodule within the pancreatic head, indicating the tumor (white arrow). The white arrowhead, hollow arrowhead and curved arrow indicates the duct of Santorini, the duct of Wirsung and the common bile duct, respectively. (b) Axial portal venous phase image, the tumor nodule showed quick wash-out of contrast agent and became only slightly hyper-enhancing compared to the adjacent pancreatic parenchyma. (c) Reconstructed coronal early arterial phase image. Besides the hyper-enhancing tumor nodule (white arrow), the duct of Santorini (white arrowhead) and the duct of Wirsung (hollow arrowhead) are more clearly visible, with the duct of Santorini in close proximity to the tumor. CT = computed tomography, pNETs = pancreatic neuroendocrine tumors.

**Figure 2.**
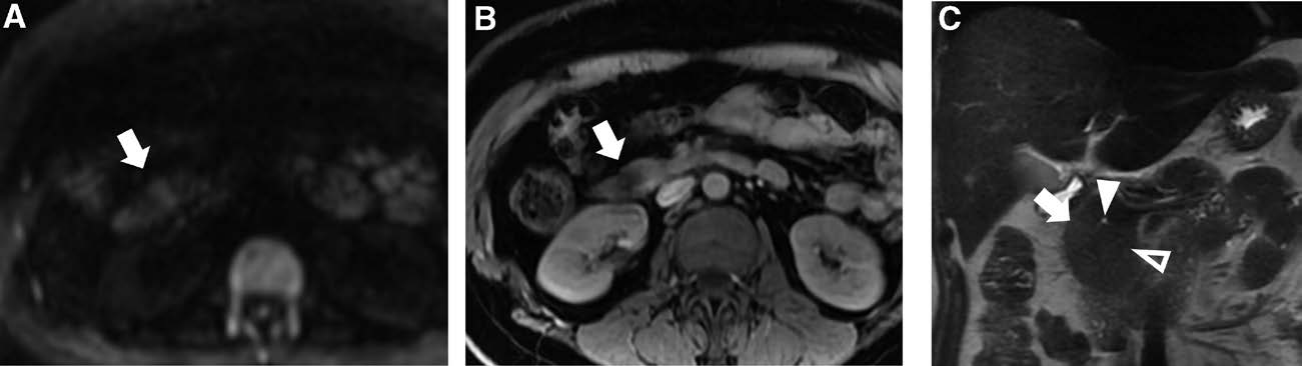
Pre-operative contrast-enhanced abdominal MRI. (a) Diffusion-weighted imaging (DWI) revealed a high signal intensity (SI) nodule in the pancreatic head (white arrow). (b) Fat-saturated T1-weighted imaging (fs-T1WI) revealed the tumor as a low-SI nodule (white arrow). (c) On coronal T2-weighted imaging (T2WI), the tumor was slightly hyper-intense compared to the pancreas, which is barely visible (white arrow). The white arrowhead and the hollow arrowhead indicates the duct of Santorini and the duct of Wirsung, respectively. MRI = magnetic resonance imaging.

In 2018, a 49-year-old woman experienced dizziness, palpitations, blurred vision, sweating, nausea, and vomiting shortly after lunch. She later lost consciousness and suffered from retrograde amnesia; all symptoms completely resolved approximately 40 minutes after onset. No incontinence, pallor, or severe headaches were observed. The patient soon underwent a blood glucose test which was reported 2.38 mmol/L. Magnetic resonance imaging of the head revealed no particular findings. However, similar symptoms appeared recurrently, mostly before meals, and were accompanied by unconsciousness. Symptoms can be relieved by feeding with water. The lowest recorded blood glucose was 1.5 mmol/L. Three months before she was admitted to our hospital, similar symptoms were observed more frequently, and night onset became increasingly common. Laboratory test results: urine acetone bodies (-), INS 6.6 uU/mL, C-P 1.56 ng/mL, GAD 1.18 U/L (0–1), IGF-1 178 ng/mL (94–252), GH 0.1 ng/mL (0.06–5), HbA1c 5.1%, ACTH (8 AM), 14.8 pg/mL (7.0–61.1), ACTH (4 PM) 11.8 pg/mL, ACTH (0 AM) 7.49 pg/mL, blood cortisol (8 AM) 10.5 ug/dL (5–25), blood cortisol (4 PM) 2.18 ug/dL, blood cortisol (0 AM) 2.03 ug/dL. During the course of the illness, the patient denied expulsion of urinary stones, gross hematuria, bone fracture, bone pain, narrowed vision, moon face, buffalo hump, striae, personality change, countenance change, acromegaly, and tooth loss. The patient also denied insulin administration and oral antidiabetic agents. No obvious abnormal changes were observed in the liver, renal, and thyroid function tests, insulin autoantibody, insular cellular antibody, HbA1c, ferritin, and cancer markers. Unsurprisingly, contrast-enhanced abdominal CT and magnetic resonance imaging revealed a slightly enhanced nodule (1.1 × 1.64 × 1.35 cm) at the pancreatic head (Figs. 1a and b and 2a and b). There was no evidence of pituitary or parathyroid abnormalities. In conclusion, the patient was a middle-aged woman with a chronic illness course, during which she experienced repetitive dizziness, palpation, and unconsciousness, which are likely to be symptoms of hypoglycemia. Her blood glucose level was frequently <2.8 mmol/L. Symptoms can be relieved by glucose intake or infusion. The collection of symptoms perfectly matches the “Whipple Triad.” Analysis of the medical history, in association with positive imaging and laboratory findings, suggested a clinical diagnosis of insulinoma. However, a reconstructed CT image revealed that although the tumor was >3 mm from the duct of Wirsung, it was extremely close to the duct of Santorini, at a distance of approximately 1 mm (Figs. 1a and c and 2c). According to our previous experience, the risk of injury to the duct in Wirsung is low; thus, stenting is not imperative. Laparoscopic pancreatic enucleation of the tumor was routinely performed.

**Figure 3.**
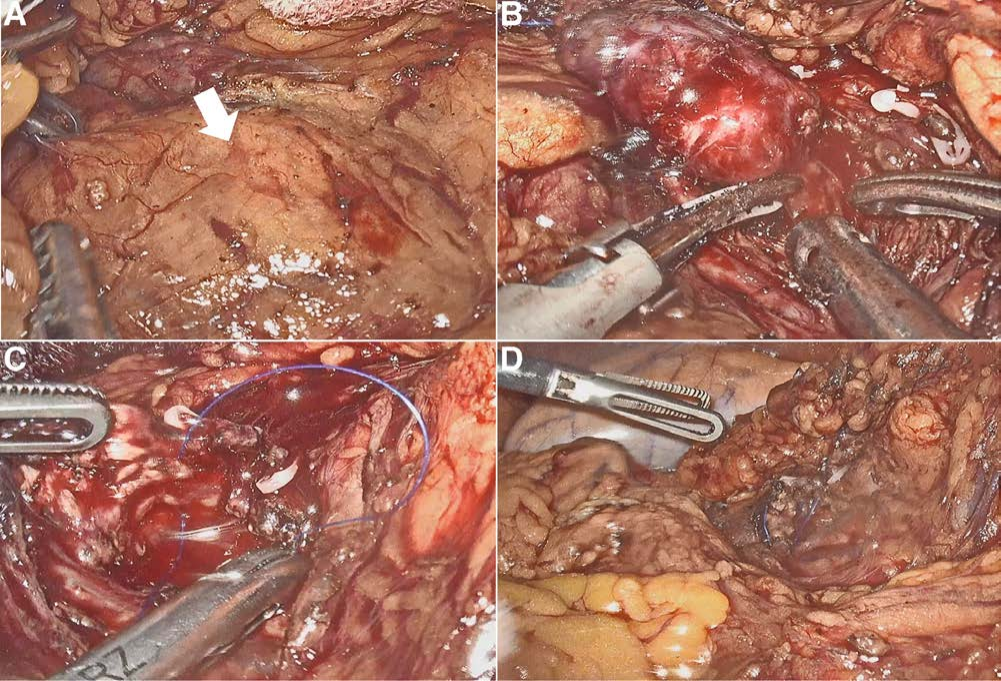
Intraoperative view of enucleation of the insulinoma at the head of the pancreas. (a) Head of the pancreas with intact capsule. White arrow highlights the region where the tumor is buried. (b) The tumor that has been mostly separated from the pancreatic parenchyma. (c) Wound of the pancreas after the tumor has been removed. (d) Wound of the pancreas that has been sutured.

**Figure 4.**
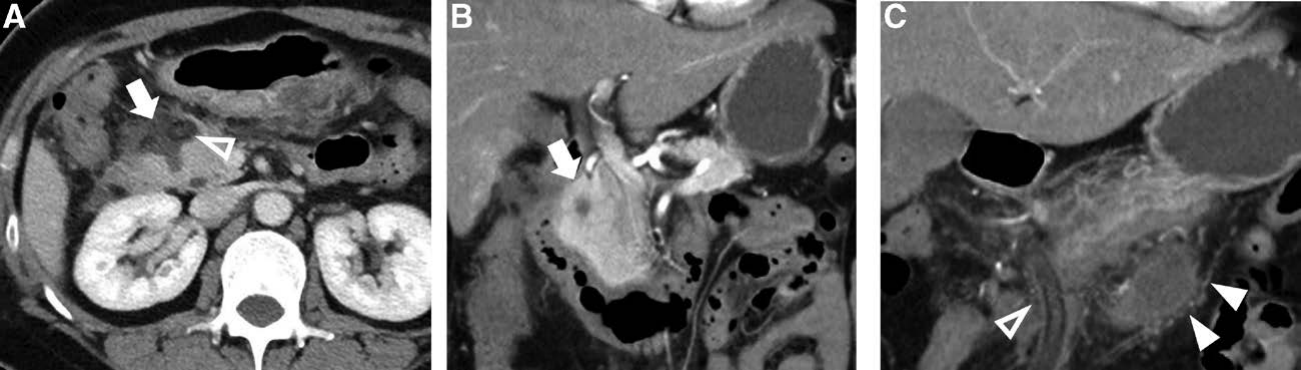
Post-operative contrast-enhanced abdominal-pelvic CT (POD 7). (a) Axial portal venous phase image. Focal parenchyma defect (white arrow) was revealed where the tumor originally situated, with effusion and draining tube in situ (hollow arrowhead). (b and c) Coronal portal venous phase image showing the parenchyma defect (white arrow) as the result of tumor enucleation. The draining tube was in situ (hollow arrowhead), and there was fluid accumulation with peripheral fat stranding on the caudal side of the pancreas, indicating post-operative fistula. CT = computed tomography, POD = postoperative day.

**Figure 5.**
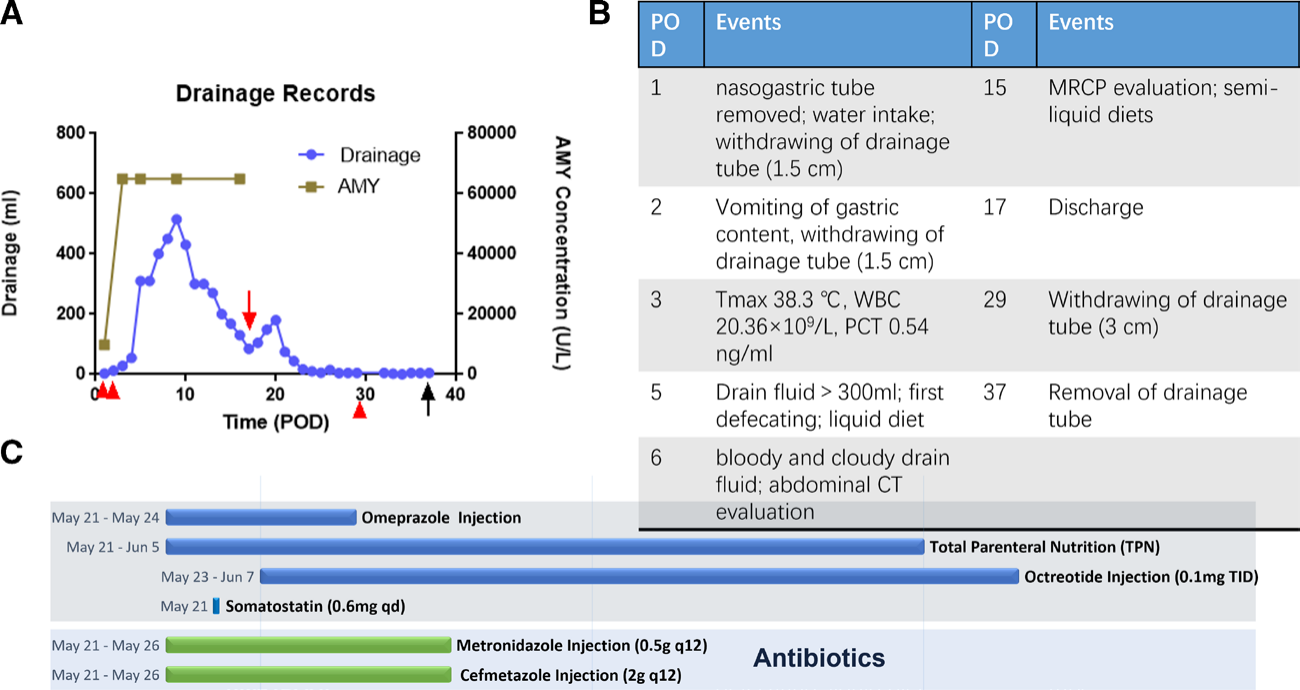
Clinical records of the patient. (a) Detailed records of drain fluid amount and AMY measurements. Day of discharge (red arrow; POD17), days to adjust the drainage tube position (red arrowhead; POD1, 2, 29) and the day to withdraw the tube (black arrow; POD 37) are highlighted. (b) Relevant events during the patient’s hospitalization. (c) A timeline of major postoperative management of the patient. AMY = amylase, POD = postoperative day.

**Figure 6.**
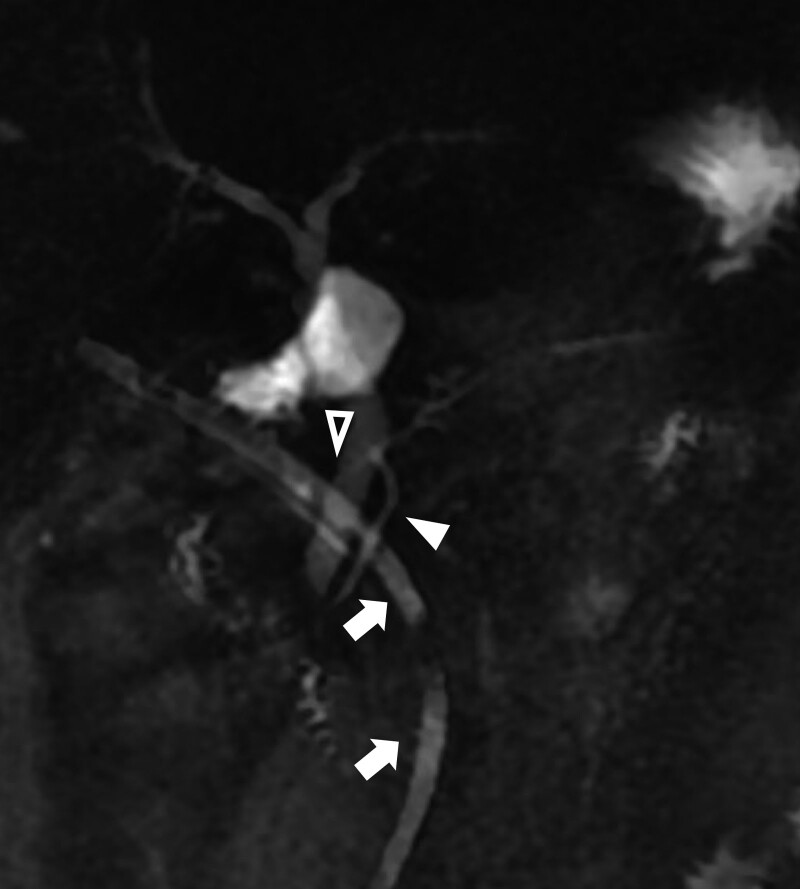
Post-operative MRCP (POD 15). The draining tube is in situ (white arrow), and the ducts of Santorini (hollow arrowhead) and Wirsung (white arrowhead) are both visible. Intersection of the duct of Wirsung and the dorsal duct is highlighted (red arrowhead). Surgical damage to the duct of Santorini, which was almost inevitable due to its path, was considered the cause of post-operative fistula. CT = computed tomography, MRCP = magnetic resonance cholangiopancreatography, POD = postoperative day.

After induction of anesthesia, pneumoperitoneum was established. General exploration of the abdominal cavity revealed no abnormalities of the liver or other organs. The gastrocolic and duodenocolic ligaments were divided to expose the pancreatic head and neck. The pancreatic texture was normal. The hepatocolic ligament was cleaned to further expose the duodenum and pancreatic head. The target lesion was localized using intraoperative ultrasonography. The tumor was completely endophytic. The capsules of the pancreas and sublobes were opened. After cleaning the aortic arch anterior to the pancreatic head, the tumor was partially visualized within the arch. The head branch of the gastroduodenal artery was suspended to expose the tumor underneath. The tumor was gently suspended using stitches, carefully separated from the pancreatic parenchyma, and completely resected (Fig. 3a–d). Investigation of the pancreatic wound surface revealed no overt leakage of pancreatic juice. Enucleation of the endophytic insulinoma was performed smoothly, and a drainage tube was placed near the pancreatic enucleation wound. Postoperatively, the blood glucose level of the patient returned to the reference range with insulin supplementation. During the first 2 days after surgery, the amount of daily abdominal drainage was <20 mL, and effusion of light-yellow tissue fluid out of the pancreatic defect was observed. Meanwhile, the patient did not have any special complaints, and his serum biochemical levels and coagulative index were generally normal. A sudden rise in abdominal drainage (310 mL) was recorded on POD5. The drain fluid amylase reported >65,000 U/L. An abdominal CT scanning on POD7 revealed local fluid accumulation with peripheral fat stranding on the caudal side of the pancreas, indicating POPF (Fig. 4a–c). We also prolonged the total parenteral nutrition and administration of somatostatin analogs (Fig. 5b and c). The amount of drained fluid remained over 300 mL until POD10, after which it gradually decreased (Fig. 5a). To pinpoint the exact site of injury that led to POPF, we performed MRCP on POD15 to evaluate the integrity of the pancreatic duct. Unsurprisingly, the duct of Wirsung remained undamaged, whereas a breakpoint of the duct of Santorini was visible, which was likely to be responsible for the fistula (Fig. 6). Considering that the patient had a restored and tolerated semi-liquid diet, and the amount of drain fluid was under 100 mL and constantly decreasing, the patient was discharged on POD19 with a drainage tube retained. We asked her to record and report the daily amount of drainage until the drainage tube was removed at our outpatient service on POD38. She did not complain of any discomfort.

## 3. Discussion

A few crucial factors must be considered before the enucleation of small pancreatic tumors. First, the tumor must exhibit either benign or low-grade features. For example, the boundary between the tumor capsule and pancreatic parenchyma is clearly distinct on preoperative imaging evaluation, with no evidence of local invasion, lymph node infiltration, or distant metastasis. Second, despite the disputation, a close association between the tumor and the pancreatic duct implies a high risk of pancreatic duct injury and POPF. A retrospective case-control study containing 60 patients undergoing standard enucleation or deep enucleation showed that although enucleation is a safe procedure for small benign tumors >3 mm distant from the MPD, careful evaluation is required when the distance is ≤3 mm because of the markedly increased rate of POPF and other complications.^[11]^ Similar conclusions have been proven by other groups, and the distance threshold is congruously between 2 and 3 mm.^[10,16]^ Notably, our previous dataset containing 161 patients undergoing insulinoma enucleation revealed that a tumor-duct distance ≤2 mm is a determinate risk factor for POPF, and pancreatic stenting can significantly lower the risk of POPF.^[12]^ In fact, most surgeons agree that a 3-mm-distance between the tumor and the MPD is generally considered safe during enucleation, efficiently avoiding POPF, and more importantly, clinically relevant POPF (Table 1). However, some groups have provided opposing evidence to support that the distance between the tumor and duct is not associated with the risk of POPF (Table 1).^[17,18]^ Despite the current controversial conclusions, auxiliary measures such as intraoperative ultrasonography are helpful and sometimes mandatory to explore the exact spatial relationship between the tumor and the duct before determining the type of surgery, especially in cases where the tumor-duct distance is small or indeterminate based on pre-operative imaging data.^[19,20]^

**Table 1:** List of literature discussing the risk of POPF and tumor-duct distance.

Disease	Type of dataset	Original size	Procedure	CR-POPF rate	Conclusion	References
pNETs, SPCNs, SCAs, MCNs, and BD-IPMNs	Retrospective, case-control study	60, single-center	DE (30), SE (30)	DE = 73.3%, SE = 30%	Tumor-duct distance ≤ 3 mm is a risk factor	Heeger et al (2014)
pNETs, MCNs, SCAs, renal metastatic cancer	Retrospective, cohort study	52, multi-center	DE (6), SE (8)	DE = 60%, SE = 19%	Tumor-duct distance ≤ 2 mm is a risk factor	Brient et al (2012)
Cystic or solid tumors	Retrospective	56, single-center	DE (11), SE (29)	DE = 72%, SE = 31%	Tumor-duct distance ≤ 3 mm is risk factor	Jin et al (2016)
Insulinoma	Retrospective	161, single-center	DE (44), SE (37)	DE = 71.4%, SE = 25.7%	Tumor-duct distance ≤ 2 mm is risk factor	Xu et al (2021)
Cystic or solid tumors	Prospective, cohort study	95, single-center	DE (21), SE (26)	DE = 50%, SE = 50%	Tumor-duct distance ≤ 2 mm is not a risk factor	Duconseil et al (2018)
Cystic or solid tumors	Prospective	166, single-center	DE (67), SE (62)	DE = 70%, SE = 67%	Tumor-duct distance ≤ 2.2 mm is not a risk factor	Strobel et al (2015)

BD-IPMN = intraductal papillary mucinous cystic neoplasms, CR-POPF = clinically relevant POPF, DE = deep enucleation, MCNs = mucinous cystadenoma, pNETs = pancreatic neuroendocrine tumors, SCAs = serous cystadenoma, SE = standard enucleation group, SPCNs = solid papillary cystic neoplasms.

In this case, a 49-year-old female patient was diagnosed with functional pancreatic insulinoma, with no special symptoms, manifestations, comorbidities and chronic conditions. Base on the preoperative imaging data, the tumor was benign was far away from the MPD (>3 cm) and a stent at the MPD was not placed. No evident leakage of pancreatic fluid was observed during surgery. The amount of abdominal drainage fluid was low during POD1-4. However, we observed a sudden jump in the volume of drainage on POD5 with no evident cause. Two unobservable reasons could have contributed to this. First, the patient was restored to an oral liquid diet on POD5, which is a direct and strong stimulus for pancreatic liquid secretion. Second, we observed a gradual and minor increase in cloudy yellow drain fluid on POD1-4, while the fluid suddenly turned flocculent POD5 and after, and large flocs, likely to be the products of fat or tissue degradation by leakage of pancreatic enzymes, were continuously visible a few days later. The inner opening of the tube can be almost blocked on POD1-4, and suddenly becomes unobstructed due to the expulsion of certain flocs due to increased local pressure. For this patient, the POPF was grade B, as defined by changes in clinical management, such as persistent drainage, according to the definition proposed by the International Study Group of Pancreatic Fistula, and lasted a month before removal of the drainage tube. Since postoperative MRCP revealed perfect integrity of the duct of Wirsung and the dorsal duct, while the duct of Santorini was interrupted near the minor papilla, the cause of POPF was explicit. Retrospectively speaking, although it was safe to discharge the patient when the amount of abdominal drainage decreased even before POD17, the patient had grade B POPF.

Though mild POPF was expected due to the close relationship between the tumor and the accessory pancreatic duct (APD), the risk of grade B/C POPF was presumably low before the surgery. A detailed retrospective MDT discussion proposed 2 main reasons for the occurrence of POPF in this case. First, the tumor was located right anterior to the duct of Santorini based on our pre-operative 3D reconstruction of CT images, and the tumor-duct distance was only about 1 mm. A minor deviation of the enucleation depth will lead to injury of the APD. More importantly, the thickness of the APD indicates that it may accounts for a considerable portion of pancreatic fluid drainage, though not quantifiable. Although the patient recovered with prolonged somatostatin use and drain tube carrying. Two questions were necessary to discuss.

**Figure 7.**
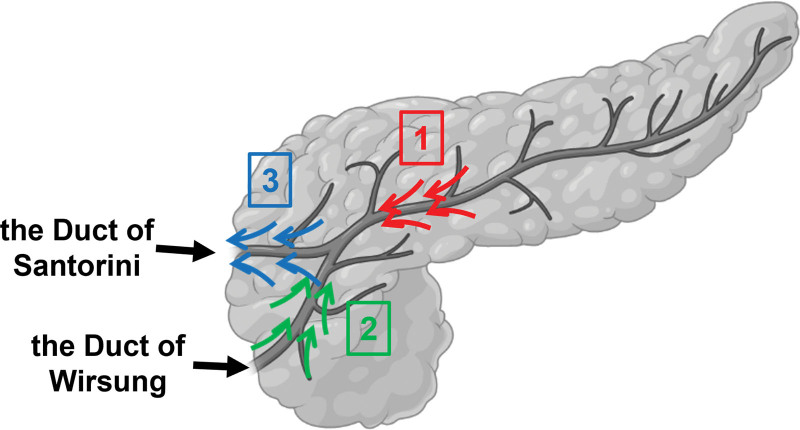
Possible directions of pancreatic fluid drainage during POPF caused by injury of the duct of Santorini. Direction 1: Normal drainage of dorsal duct. Direction 2: Regurgitation of the duct of Wirsung. Direction 3: Normal drainage of duct of Santorini. POPF = post-operative pancreatic fistula.

First, would pancreatic stenting be beneficial in this case? Studies discussing the association between the relative position of the tumor and the APD and POPF are lacking. From a support perspective, pancreatic stenting guarantees adequate drainage of the MPD, decompresses the entire ductal system, and reduces leakage out of the pancreatic wound during POPF. However, the benefit of MPD stent in the case of APD injury remain undefined clinically. It remains unknown how much stenting can improve the clinical outcome, considering that the pancreatic fluid leakage can come from multiple directions, while stenting of the duct of Wirsung only deals with drainage from direction 1 and perhaps, 2, partially (Fig. 7). Particularly in this case, the intersection of the dorsal duct and the duct of Wirsung is not perfectly smooth, with increased difficulty in stenting the duct at a proper position (Fig. 6). Moreover, the possibility of iatrogenic injury to the pancreas and nearby organs during ERCP is another reason to deny the use of pancreatic stents. Second, if the risk of serious POPF is presumably high and the benefit of pancreatic duct stenting is uncertain, would enucleation be the best option for this patient? Limited clinical evidence can supported an answer of this question.

In conclusion, we have gained valuable experience in the management of this patient. First, thin-layer (1 mm) abdominal CT scanning and reconstruction can clearly reveal the spatial relationship between the tumor and the major branches of the pancreatic duct, which is invisible in regular CT images unless dilated. Both the APD-tumor relationship and the MOD-tumor relationship need to be considered since a congenitally wide APD can be clinically relevant. Second, tumors located at the pancreatic head need extra attention during surgery since injury to the duct of Santorini is clinically significant and may not be visible during surgery. Third, in the absence of pre-operative and remedial pancreatic duct stenting or other special interventions, POPF caused by injury to the duct of Santorini may be completely self-limiting. However, the exact benefit of pre-operative stenting and possible interventions when POPF occurs in cases of expected injury of the duct of Santorini still needs to be evaluated by well-designed clinical trials. Other techniques that specifically lower the risk of POPF related to injury of the duct of Santorini are warranted.

## Author contributions

**Conceptualization:** Qiang Xu, Yupei Zhao.

**Data curation:** Tiantong Liu, Xi Zou.

**Formal analysis:** Tiantong Liu.

**Investigation:** Tiantong Liu, Qiang Xu, Xi Zou, Liang Zhu.

**Methodology:** Tiantong Liu.

**Project administration:** Tiantong Liu, Yupei Zhao.

**Resources:** Tiantong Liu.

**Software:** Tiantong Liu.

**Supervision:** Qiang Xu.

**Validation:** Tiantong Liu, Xi Zou.

**Visualization:** Tiantong Liu.

**Writing – original draft:** Tiantong Liu.

**Writing – review & editing:** Qiang Xu.
